# Soluble ANPEP Released From Human Astrocytes as a Positive Regulator of Microglial Activation and Neuroinflammation: Brain Renin–Angiotensin System in Astrocyte–Microglia Crosstalk

**DOI:** 10.1016/j.mcpro.2022.100424

**Published:** 2022-10-08

**Authors:** Jong-Heon Kim, Ruqayya Afridi, Eunji Cho, Jong Hyuk Yoon, Yong-Hyun Lim, Ho-Won Lee, Hoon Ryu, Kyoungho Suk

**Affiliations:** 1 Brain Science & Engineering Institute, Kyungpook National University, Daegu, Republic of Korea; 2 Department of Biomedical Science, School of Medicine, Kyungpook National University, Daegu, Republic of Korea; 3 Neurodegenerative Diseases Research Group, Korea Brain Research Institute, Daegu, Republic of Korea; 4 Center of Self-Organizing Software-Platform, Kyungpook National University, Daegu, Republic of Korea; 5 Department of Neurology, Kyungpook National University Chilgok Hospital, School of Medicine, Kyungpook National University, Daegu, Republic of Korea; 6 Center for Neuromedicine and Neuroscience, Brain Science Institute, Korea Institute of Science and Technology, Seoul, Republic of Korea; 7 Boston University Alzheimer's Disease Center and Department of Neurology, Boston University School of Medicine, Boston, Massachusetts, USA

**Keywords:** brain renin–angiotensin system, soluble aminopeptidase N, glial crosstalk, microglia, astrocyte, secretome, neuroinflammation, ACE, angiotensin-converting enzyme, AD, Alzheimer’s disease, Ang, angiotensin, ANPEP, alanyl aminopeptidase, AT1R, angiotensin type 1 receptor, BUADC, Boston University’s Alzheimer’s Disease Center, cDNA, complementary DNA, CM, conditioned medium, CNS, central nervous system, CSF, cerebrospinal fluid, GO, Gene Ontology, GPCR, G protein–coupled receptor, i.c.v., intracerebroventricular, IFN-γ, interferon gamma, IL-1β, interleukin 1β, IRB, Institutional Review Board, KEGG, Kyoto Encyclopedia of Genes and Genomes, LPS, lipopolysaccharide, MasR, Mas receptor, MS, mass spectrometry, RAS, renin–angiotensin system, sANPEP, soluble form of aminopeptidase N, TNF-α, tumor necrosis factor alpha

## Abstract

Astrocytes are major supportive glia and immune modulators in the brain; they are highly secretory in nature and interact with other cell types *via* their secreted proteomes. To understand how astrocytes communicate during neuroinflammation, we profiled the secretome of human astrocytes following stimulation with proinflammatory factors. A total of 149 proteins were significantly upregulated in stimulated astrocytes, and a bioinformatics analysis of the astrocyte secretome revealed that the brain renin–angiotensin system (RAS) is an important mechanism of astrocyte communication. We observed that the levels of soluble form of aminopeptidase N (sANPEP), an RAS component that converts angiotensin (Ang) III to Ang IV in a neuroinflammatory milieu, significantly increased in the astrocyte secretome. To elucidate the role of sANPEP and Ang IV in neuroinflammation, we first evaluated the expression of Ang IV receptors in human glial cells because Ang IV mediates biological effects through its receptors. The expression of angiotensin type 1 receptor was considerably upregulated in activated human microglial cells but not in human astrocytes. Moreover, interleukin-1β release from human microglial cells was synergistically increased by cotreatment with sANPEP and its substrate, Ang III, suggesting the proinflammatory action of Ang IV generated by sANPEP. In a mouse neuroinflammation model, brain microglial activation and proinflammatory cytokine expression levels were increased by intracerebroventricular injection of sANPEP and attenuated by an enzymatic inhibitor and neutralizing antibody against sANPEP. Collectively, our results indicate that astrocytic sANPEP–induced increase in Ang IV exacerbates neuroinflammation by interacting with microglial proinflammatory receptor angiotensin type 1 receptor, highlighting an important role of indirect crosstalk between astrocytes and microglia through the brain RAS in neuroinflammation.

## Highlights

• Human astrocyte secretome analysis following stimulation with proinflammatory factors.
• Human astrocytes release components of the brain renin–angiotensin system.
• Soluble form of aminopeptidase N positively regulates microglial activation.
• Astrocyte–microglia crosstalk occurs by activating the brain renin–angiotensin system during neuroinflammation.

## Graphical Abstract

**Figure:**
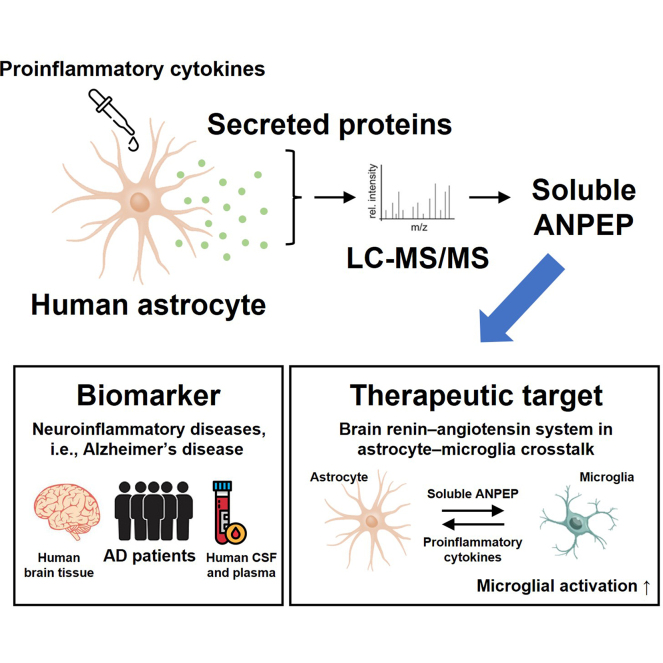

## In Brief

Understanding the astrocyte secretome may provide insights into the regulatory mechanisms of neuroinflammation through astrocyte-mediated communication. In this study, we found that human astrocytes release the soluble form of aminopeptidase N as a component of the brain renin–angiotensin system, positively regulating microglial activation. Our findings suggest the diagnostic and therapeutic potential of soluble form of aminopeptidase N in neuroinflammatory diseases.

Astrocytes are the major glial cell type of the central nervous system (CNS) and play important roles in brain homeostasis, such as metabolic and trophic support; ion, fluid, and neurotransmitter balance regulation; and blood–brain barrier maintenance. These cells also play an important role in controlling innate immune responses in the CNS (1). Astrocytes release various secretory mediators that act as signaling molecules and interact with other cell types to regulate inflammatory responses. For example, astrocytes crosstalk with microglia to orchestrate neuroinflammation, and this interglial communication is maintained in part *via* the secreted molecules, such as cytokines, chemokines, other inflammatory mediators, and tissue damage–associated molecules. Therefore, a better understanding of this crosstalk will provide insights into the regulatory mechanisms of neuroinflammation. Moreover, proteomic analysis of glial-secreted molecules could provide critical clues for deciphering the interglial crosstalk.

The renin–angiotensin system (RAS) is a well-known hormonal system that regulates blood pressure and fluid homeostasis. The RAS hormone angiotensinogen is generated by the liver and circulates in plasma, and angiotensinogen is cleaved by renin into angiotensin (Ang) I, a precursor of Ang II. The effector peptide Ang II is formed by sequential cleavage of Ang I by angiotensin-converting enzyme (ACE). Ang II can be degraded to Ang III and Ang IV by various aminopeptidases, including aminopeptidase A, aminopeptidase N, and dipeptidyl peptidase-4, and it can also be converted to Ang (1, 2, 3, 4, 5, 6, 7) by ACE2, an isoform of ACE. Ang II and Ang (1, 2, 3, 4, 5, 6, 7) are ligands of the angiotensin type 1 receptor (AT1R) and Mas receptor (MasR), respectively, and they exert opposing biological actions (2).

Initially, RAS was considered part of the endocrine system; however, the local form of RAS in the brain was also discovered later. Mounting evidence indicates that the brain RAS is strongly involved in neuroinflammation and actively participates in various neurological conditions (3, 4, 5). For example, microglial activation and exacerbation of oxidative stress are closely related to the brain RAS (6). Ang II is the most important effector peptide of the brain RAS and has been suggested to be the main neuroinflammatory trigger (7). As Ang II can be rapidly cleaved into Ang III and Ang IV by angiotensinase, these metabolites may also play a key role in brain pathology (8, 9). For example, brain Ang III controls hypertension (10) and contributes to Alzheimer’s disease (AD) pathogenesis (11). Ang IV is associated with cerebrovascular dysfunction as well as memory deficits and recovery (12). In terms of neuroinflammation, long-term Ang IV infusion suppresses inflammation in injured brain, thereby counteracting the effects of proinflammatory Ang II (13); however, acute treatment with Ang IV activates NF-κB to induce the expression of proinflammatory genes, thereby enhancing neuroinflammation (14, 15).

The RAS acts through various angiotensin receptor subtypes, including AT1R, AT2R, MasR, and AT4R. The ACE/Ang II/AT1R pathway has been shown to exert proinflammatory effects in microglia, leading to neuroinflammation (16, 17, 18), whereas an excessive inflammatory response can be counterbalanced by the ACE/Ang II/AT2R and ACE2/Ang (1, 2, 3, 4, 5, 6, 7)/MasR axes (19, 20). Ang IV can bind to anti-inflammatory AT4R, and at high concentrations, it can also bind to proinflammatory AT1R (21). Therefore, local angiotensinase activity and the Ang receptor distribution may be crucial for determining Ang IV function.

Alanyl aminopeptidase (ANPEP), also known as aminopeptidase N, is an angiotensinase that hydrolyzes the N-terminal arginine of Ang III to generate Ang IV. ANPEP has also been identified as type II transmembrane protein CD13, which is a widely expressed moonlighting ectoenzyme (22). This protein also exists in shed and secreted soluble forms (23). Because the soluble form of ANPEP (sANPEP) has a conserved and extracellular catalytic domain, it is fully active enzymatically (24). The aberrant expression of sANPEP has been observed in various diseases. For example, sANPEP has been suggested as a biomarker for early onset chronic graft-*versus*-host disease (25), malignant tumors (26), and rheumatoid arthritis (27). Moreover, sANPEP has been suggested as a therapeutic target in various inflammatory diseases (27, 28), including neuroinflammation.

In this study, we identified the upregulation of brain RAS–associated proteins by conducting a secretome analysis of human astrocytes. Enhanced secretion of sANPEP from activated astrocytes under inflammatory conditions increases Ang IV levels *via* Ang III-cleaving enzymatic activity, which may facilitate proinflammatory microglial activation and neuroinflammation by interacting with the microglial proinflammatory receptor AT1R. Our findings suggest a novel mechanism of astrocyte–microglia crosstalk through the brain RAS.

## Experimental Procedures

### Reagents

Recombinant human tumor necrosis factor alpha (TNF-α), interleukin 1β (IL-1β), interferon gamma (IFN-γ), sANPEP, and Ang IV and mouse sANPEP were purchased from R&D Systems. Lipopolysaccharide (LPS) from *Escherichia coli* 0111:B4 and human and mouse Ang III peptides were purchased from Sigma–Aldrich. Bestatin was purchased from Toris. Neutralizing monoclonal antibodies against human ANPEP (clone: WM15) (29) and mouse ANPEP (clone: SL13) (30) were purchased from BioLegend and Millipore, respectively.

### Cell Culture and Animal Care

#### Cell Culture

Human astrocytes were purchased from ScienCell and grown in poly-l-lysine–coated culture flasks in astrocyte medium (ScienCell) according to the manufacturer’s instructions. Human microglial cells (HMC-3 cell line) were purchased from the American Type Culture Collection. The cells were cultured in minimum essential medium (Gibco, Thermo Fisher Scientific) supplemented with 10% fetal bovine serum (Merck KGaA) and antimicrobials (100 U/ml penicillin and 100 μg/ml streptomycin; Gibco, Thermo Fisher Scientific). All cells were cultured under mycoplasma-free conditions, maintained at 37 °C in a 5% CO_2_ atmosphere, and subcultured when they reached 80 to 90% confluence. We used human astrocytes and microglial cells at passages 3 to 5.

#### Animal Care Procedures

Male C57BL/6 mice (8 to 9 weeks old) were purchased from Samtako Bio. 5xFAD mice (3–8 months of age) overexpressing the K670N/M671L (Swedish mutation), I716V (Florida mutation), and V717I (London mutation) mutations in human amyloid precursor protein (isoform 695), as well as the M146L and L286V mutations in human presenilin-1, were kindly provided by the Korea Brain Research Institute. All mice were housed in groups of three to five per cage under specific pathogen-free conditions and a 12-h/12-h light/dark cycle. All animal experiments were approved by Kyungpook National University Animal Care Committee (KNU 2022-0015), and care was taken to minimize animal suffering.

### Nitrite Concentration Quantification

Cells were stimulated with TNF-α, IL-1β, and IFN-γ (10 ng/ml each) after they were transferred to 96-well plates, and nitrite (NO_2_^−^) concentration in the medium was measured to assess NO production using the Griess reaction, as described previously (31). Subsequently, 50 μl aliquots of the sample were mixed with 50 μl of Griess reagent (1% sulfanilamide/0.1% naphthylethylene diamine dihydrochloride/2% phosphoric acid) in a 96-well plate. Absorbance at 550 nm was measured using a microplate reader (Molecular Devices). NaNO_2_ was used as the standard to calculate the NO_2_^−^ concentrations.

### Preparation of Secreted Proteins From Human Astrocyte Cultures

Human astrocytes were incubated with TNF-α, IL-1β, and IFN-γ (10 ng/ml each) for 24 h. Then, the cells were washed thoroughly five times using 10 ml of Hank’s balanced salt solution without Ca^2+^ and Mg^2+^ and replenished with the fresh serum-free human astrocyte medium. After 24 h, the conditioned medium (CM) was collected, and a protease inhibitor mixture (Roche Diagnostics) was immediately added. Cell debris was removed by centrifugation at 500*g* for 10 min at 4 °C, and secreted proteins in the CM were concentrated using a Centricon filter (4 kDa molecular weight cutoff; GE Healthcare). The concentrated samples were diluted in 200 μl of PBS, and total proteins were quantified using the Bradford method (Bio-Rad).

### Secretome Analysis

#### Peptide Generation Using in-Solution Digestion

For in-solution digestion, precipitated proteins were resuspended in 40 mM NH_4_HCO_3_. After 20 min of incubation with 5 mM dithiothreitol at 56 °C, protein precipitates were treated with 10 mM iodoacetamide for 15 min in the dark at 20 to 25 °C. The protein precipitates were then treated with a 1:100 trypsin–Lys C mixture (Promega) for 12 h at 37 °C. Tryptic-digested peptides were lyophilized and desalted using a desalting column (Thermo Fisher Scientific) according to the manufacturer’s protocol.

#### Mass Analysis and Database Search

Tryptic-digested peptides were analyzed using a Q-Exactive plus hybrid quadrupole orbit-trap mass spectrometer (Thermo Fisher Scientific) interfaced with an EASY-Spray source. Chromatographic separation of peptides was achieved on an Ultimate 3000 RSLCnano System (Thermo Fisher Scientific) equipped with an Acclaim PepMap 100 (75 mm × 2 cm, 3 μm, NanoViper; Thermo Fisher Scientific) as the loading column and an EASY-Spray column PepMap RSLC C18 (75 μm × 50 cm, 2 μm; Thermo Fisher Scientific) as the separation column. Peptides were loaded from an RS autosampler and separated with a linear gradient of acetonitrile/water containing 0.1% formic acid at a flow rate of 300 nl/min. The liquid chromatography eluent was electrosprayed directly from the analytical column, and a voltage of 2.0 kV was applied *via* the liquid junction of the nanospray source. Peptide mixtures were separated using a 5% to 40% acetonitrile gradient for 40 min. This analysis method involved a full mass spectrometry (MS) scan with a range of 350 to 2000 *m/z* and data-dependent MS/MS (MS2) on the 10 most intense ions from the full MS scan. The mass spectrometer was programmed to acquire data in the data-dependent mode. The mass spectrometer was calibrated using the proposed calibration solution according to the manufacturer’s instructions. To perform a database search, tandem mass spectra were processed using the software Proteome Discoverer, version 2.3 (Thermo Fisher Scientific). Spectral data were searched against the Human UniProt database (release version 2017_06) (93,591 protein entries). The analysis workflow included four nodes: Spectrum Files (data input), Spectrum Selector (spectrum and feature retrieval), Sequest HT (sequence database search), and Percolator (peptide spectral match or peptide spectral match validation and false discovery rate analysis). All identified proteins had a false discovery rate of ≤1%, which was calculated at the peptide level. The validation was based on the *q* value. Search parameters allowed for tryptic specificity of up to two missed cleavages, with methylthio modification of cysteine as a fixed modification and oxidation of methionine as a dynamic modification. The mass search parameters for +1, +2, and +3 ions included mass error tolerances of 20 ppm for precursor ions and 0.6 Da for fragment ions. Only proteins with two or more unique peptide identifications from all three replicates were considered. Differentially expressed proteins were quantified using spectral counts and defined by the following cutoff criteria: increased proteins, an average fold change ≥0.5 (on the log_2_ scale); decreased proteins, an average fold change ≤0.5 (on the log_2_ scale); and *p* value >1.3 (on the −log_10_ scale). Data were evaluated using several bioinformatics tools for pathway enrichment analyses: Gene Ontology (GO) biological processes and Kyoto Encyclopedia of Genes and Genomes (KEGG) pathways using the ClueGo plug-in (Cytoscape software v3.7.1, supported by National Institute of General Medical Sciences [NIGMS]).

### RNA Analysis

For conventional or real-time reverse transcription–PCR, total RNA was isolated from cells or brains using the QIAzol reagent (Qiagen) following the manufacturer’s instructions. Reverse transcription and PCR amplification were performed using a C1000 Touch thermal cycler (Bio-Rad) with specific primer sets. *GAPDH* was used as an internal control. Subsequently, 100 μg of RNA was used for complementary DNA (cDNA) preparation using an iScript cDNA Synthesis Kit (Bio-Rad) according to the manufacturer’s protocol, and the cDNA from each sample was amplified using real-time PCR with the iQ SYBR Green Supermix (Bio-Rad). RNA levels were normalized to *GAPDH* mRNA levels, which served as a reference. The PCR cycling conditions were as follows: denaturation for 3 min at 95 °C and 40 cycles of amplification for 15 s at 95 °C, 15 s at 60 °C, and 20 s at 70 °C, followed by 30 s at 72 °C. For the melting curve analysis, data were collected from 33 cycles (6 s each) as the temperature increased from 60 °C to 92 °C (set-point temperature increased after cycle 2 by 1 °C). The nucleotide sequences of the primers were based on published cDNA sequences (supplemental Table S1). Total RNA was extracted using the Illumina TruSeq RNA sample preparation kit and was sequenced on the Illumina HiSeq2000 platform. Raw reads were aligned to the human genome (GRCh37.p13) using the STAR 2-pass method (32). Duplicated reads were removed using Picard MarkDuplicates, and filtered reads were further processed for variant calling using GATK, including Insertion/Deletion Realignment, Base Quality Score Recalibration, and HaplotypeCaller. According to the Ensembl gene set, we used HTSeq to count the reads aligned to each gene (33).

### Clinical Samples

A total of 16 autopsy cases were examined from the Boston University’s Alzheimer’s Disease Center (BUADC), including individuals with and without cognitive impairment who underwent annual cognitive evaluations using the National Alzheimer’s Disease Coordinating Center Uniform Data Set protocol (34). Neuropathological processing of control and AD human brain samples was performed according to procedures previously established by the BUADC (35, 36). Institutional review board (IRB) approval for ethical permission was obtained from the BUADC. This study was reviewed by the Boston University School of Medicine IRB (protocol H-28974). The next of kin provided informed consent for participation and brain donation. The study was performed in accordance with the institutional regulatory guidelines and principles of the Declaration of Helsinki. Detailed information regarding the brain tissue specimens is provided in supplemental Table S2. In all cases in which AD was diagnosed at autopsy, it was also stated as the cause of death.

Human cerebrospinal fluid (CSF) and plasma samples were collected from patients who visited the Neurodegenerative Disease Center in the Kyungpook National University Chilgok Hospital (Daegu, South Korea). Prior to participation, informed consent was obtained from the patients and their caregivers. This study was approved by the IRB of Kyungpook National University Chilgok Hospital (IRB no. 2015–05-204). A total of 33 patients with AD and 22 healthy controls were enrolled in this study. Clinical, brain MRI, and neuropsychological data, including the Mini-Mental State Examination and Clinical Dementia Rating scores, were obtained for each participant. Plasma and CSF samples were collected according to the established protocols. Subjects with major medical or psychiatric issues as well as other neurological problems that could affect cognitive function were excluded. All participant characteristics are summarized in supplemental Table S3.

### ELISA

To determine the level of sANPEP in the plasma of LPS-injected mice (sacrificed 72 h after intraperitoneal injection of 1 mg/kg LPS, male) and 5xFAD mice (8 months of age, male), blood samples were collected from the vena cava using EDTA tubes and shaken gently. To separate plasma from the blood, samples were centrifuged (1500*g*, 15 min, 4 °C), and then, the plasma was transferred into a new tube. Samples (1:100) were diluted and assessed using a Mouse ANPEP ELISA Kit (MyBioSource) according to the manufacturer’s instructions. To measure the levels of human sANPEP in the clinical samples, plasma (1:10,000) and CSF (1:10) were first diluted. ELISA kits for human ANPEP (R&D Systems), TNFAIP6 (MyBioSource), MXRA8 (Cusabio), PEDF (Cloud-Clone Corp), ADAM9 (R&D Systems), and C1NH (R&D Systems) were used for the assay according to the manufacturer’s instructions. The level of human IL-1β in the conditioned media was determined using a human IL-1β ELISA Kit (R&D Systems) following the manufacturer’s instructions. The absorbance was read at 450 and 540 nm using a microplate reader (Molecular Devices).

### Mouse Model of Systemic LPS-Induced Neuroinflammation and Intracerebroventricular Injection

LPS was injected systemically as described previously (37). Mice were administered the vehicle (same volume of PBS) or LPS (1 mg/kg) i.p. For intracerebroventricular (i.c.v.) injection of sANPEP and/or monoclonal neutralization antibody, the mice were first anesthetized using isoflurane and then restrained using a stereotaxic device (Stoelting Co). Recombinant mouse sANPEP protein (3 μg per mouse) and/or neutralization antibody (6 μg per mouse) were then slowly injected (0.2 μl/min) into the left lateral ventricle using a 5 μl Hamilton syringe and microinjector (Panlab, Harvard Apparatus).

### Histology

Mice were anesthetized with ether and transcardially perfused with 4% paraformaldehyde in PBS; subsequently, their extracted brains were postfixed and cryoprotected using a 30% sucrose solution for 3 days. The fixed brains were then embedded in optimal cutting temperature compound (Tissue-Tek; Sakura Fine-Tek) and cut into 20-μm-thick sections on a cryostat. For the immunofluorescence analysis of animal tissues, frozen brain sections were permeabilized in 0.1% Triton X-100 and blocked with 1% bovine serum albumin and 5% normal donkey serum for 1 h at room temperature. The brain sections were incubated with anti-ionized calcium-binding adapter molecule 1 (Iba-1) (rabbit immunoglobulin G, 1:500 dilution; Wako) at 4 °C overnight, which was followed by incubation for 1 h at room temperature with FITC-conjugated anti-rabbit (1:200 dilution; Jackson ImmunoResearch Laboratories). Finally, the sections were mounted and counterstained with 4′,6-diamidino-2-phenylindole-containing gelatin. The intensity of Iba-1-positive cells was measured using the FIJI/ImageJ software (National Institutes of Health).

### Behavioral Tests

#### Y-Maze Test

The Y-maze test consists of a horizontal maze with three arms (length, 40 cm; width, 3 cm; and wall height, 12 cm). The tested animals were initially placed in the center of the maze, and the order (*e.g.*, ABCCAB) and number of arm entries were manually recorded over a period of 7 min for each animal. Voluntary shifts were defined as trials with entries into all three arms in sequence (*i.e.*, ABC, CAB, or BCA, but not BAB). After each test, the maze was thoroughly cleaned with water to remove any residual animal odor. The ratio of alternatives was calculated using the following equation: % alternation = ([number of alternations]/[total arm entries]) × 100. The total number of arm entries was used as an indicator of the locomotor activity. All recordings and calculations were automatically obtained using the video tracking software SMART (version 3.0, Harvard Apparatus).

#### Sucrose Preference Test

A sucrose preference test was performed as previously described using mice with free access to both water and sucrose solutions (38), and it began after 2 days of treatment with LPS and other reagents. One water bottle filled with 1% sucrose solution and another bottle filled with water were placed in the cages for 24 h. Consumption from each bottle was measured daily, and sucrose preference was expressed as follows: (Δ weight sucrose)/(Δ weight sucrose + Δ weight water) × 100.

### Statistical Analyses

Data are presented as the mean ± SEM. Comparisons between two groups were performed using either the Welch’s *t* test or unpaired nonparametric Mann–Whitney *U* test. For multiple groups, we used one-way ANOVA followed by the Tukey’s post hoc test. All statistical analyses were performed using the Prism software (version 8.0; GraphPad Software). Statistical significance was set at *p <* 0.05.

### Experimental Design and Statistical Rationale

For proteomics experiments, we have used total protein samples in the CM from human astrocyte cultures, which were either unstimulated or stimulated with TNF-α, IL-1β, and IFN-γ (10 ng/ml each) for 24 h (n = 3 replicate wells per group). Protein annotations were based on the Human UniProt database (release version 2019_07). The enriched GO-Terms were filtered for Bonferroni-corrected *p* values of <0.05. For other experiments, the statistical test and experimental details are provided in each figure legend. Data analysis and visualizations were performed in Prism software (version 8.0; GraphPad Software, www.graphpad.com).

## Results

### Secretome Analysis of Human Astrocytes

**Fig. 1:**
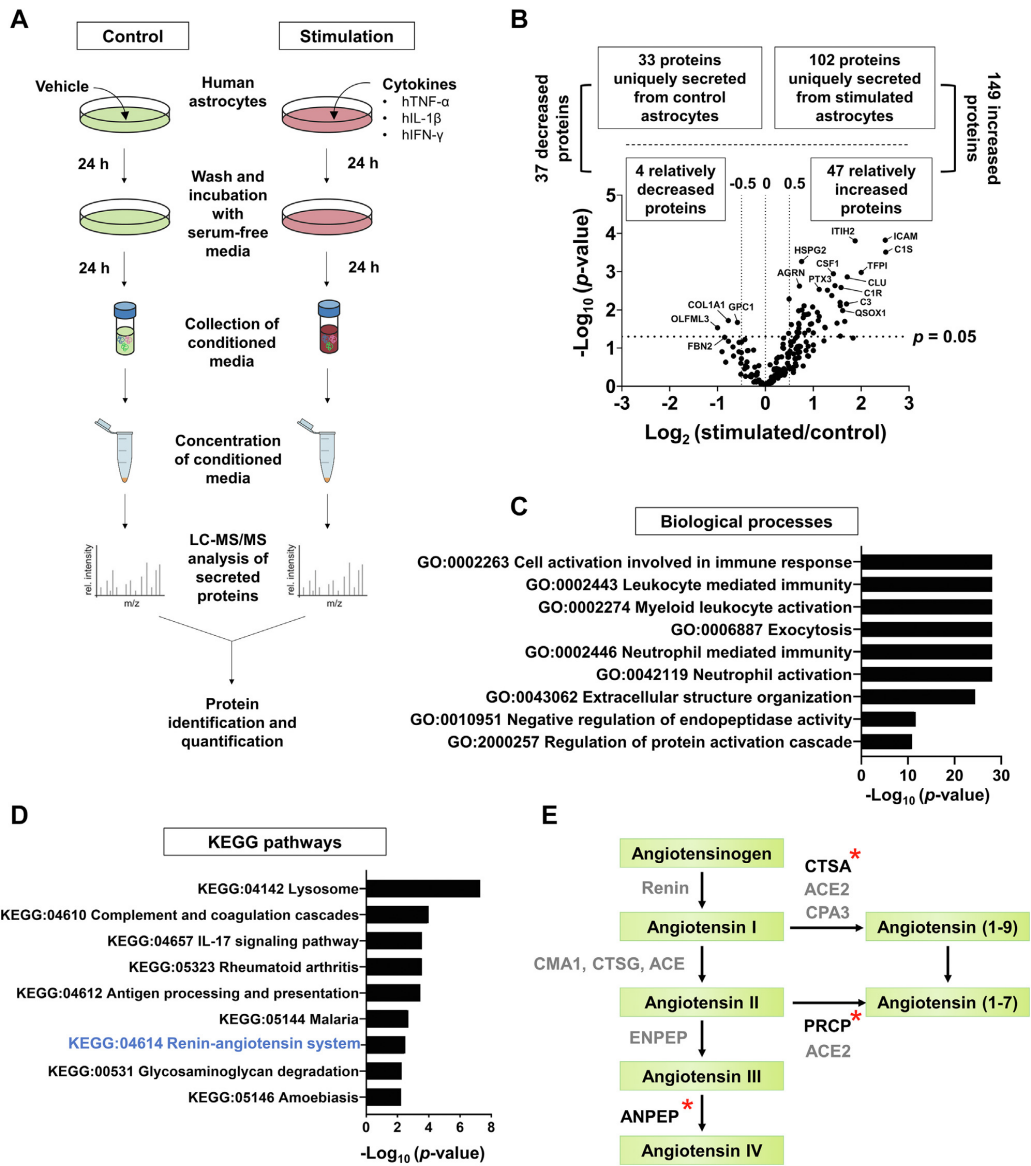
**Experimental design and proteomic analysis of human astrocyte secretome.***A*, schematic drawing of the secretome analysis procedure. Human astrocytes were stimulated with recombinant human TNF-α, IL-1β, and IFN-γ (10 ng/ml each) for 24 h and then washed thoroughly five times using 10 ml of HBSS without Ca^2+^ or Mg^2+^, and replenished with serum-free human astrocyte medium. After 24 h, the conditioned medium was concentrated by using a Centricon filter (4 kDa MWCO) and subjected to LC/MS–MS analysis. *B*, volcano plot of the −log_10_*p* value *versus* the log_2_ differential expression levels (increased >0.5, decreased <−0.5) for proteins secreted from stimulated astrocytes (stimulation) relative to unstimulated astrocytes (control). *C*, GO term enrichment analysis (biological processes) of upregulated proteins in the stimulated human astrocytes. *D*, KEGG pathways of upregulated proteins in the stimulated human astrocytes. Renin–angiotensin system is highlighted in *blue*. *E*, simplified “renin–angiotensin system” pathway. *Asterisks* indicate the upregulated proteins identified in this study. GO, Gene Ontology; HBSS, Hank’s balanced salt solution; IFN-γ, interferon gamma; IL-1β, interleukin 1β; KEGG, Kyoto Encyclopedia of Genes and Genomes; MWCO, molecular weight cutoff; TNF-α, tumor necrosis factor alpha.

For inducing inflammatory condition in human astrocytes, the cells were treated for 24 h with a cytokine mixture containing TNF-α, IL-1β, and IFN-γ (10 ng/ml each) (Fig. 1*A*). The experimental conditions for the inflammatory stimulation of human astrocytes were optimized based on NO production and the gene expression of proinflammatory cytokines (supplemental Fig. S1). The secretome of the CM of stimulated human astrocytes was analyzed using LC–MS/MS according to the outlined workflow (Fig. 1). In total, 322 proteins were identified (supplemental Fig. S2) in the astrocyte CM. Among the 322 proteins, 149 were upregulated and 37 were downregulated in astrocyte CM following inflammatory stimulation (supplemental Table S4). Our data covered 25% of the secretome of rodent astrocytes (39, 40) and 15% of that of human astrocytes (41), as reported in earlier studies (supplemental Fig. S2*B*). Also, our secretome datasets of human astrocytes provided unique secreted proteins that have not been reported previously. Upregulation or downregulation seen with LC–MS/MS was validated using ELISA for several proteins (supplemental Fig. S3). The functional distribution of the differentially expressed proteins was determined using GO biological process enrichment and KEGG pathway analyses with the ClueGo bioinformatics tool (supplemental Tables S5 and S6). The GO enrichment analysis revealed that inflammation-related functions such as “cell activation involved in immune response,” “leukocyte-mediated immunity,” “myeloid leukocyte activation,” “exocytosis,” “neutrophil-mediated immunity,” and “neutrophil activation” were significantly enriched in the CM of stimulated astrocytes, thus confirming their inflammatory activation state (Figs. 1*C* and S4*A*). The KEGG pathway enrichment analysis indicated that upregulated proteins in the activated astrocytes were related to inflammation-associated pathways such as “lysosome,” “complement and coagulation cascades,” “IL-17 signaling pathway,” and “RAS” (Figs. 1*D* and S4*B*). Downregulated proteins such as A2M, C4A, CFH, and SERPINC1 were associated with “complement and coagulation cascades,” and they were mostly anti-inflammatory proteins (42, 43, 44, 45) (supplemental Fig. S4*C*). Among the enriched biological processes and molecular pathways, we focused on the “RAS,” which was associated with several upregulated proteins such as CTSA, PRCP, and sANPEP. Particular emphasis was placed on sANPEP in a subsequent study (Figs. 1*E* and S5).

### Upregulation of sANPEP in the Body Fluid and Brain Tissue of the Neuroinflammation Model and AD Patients

**Fig. 2:**
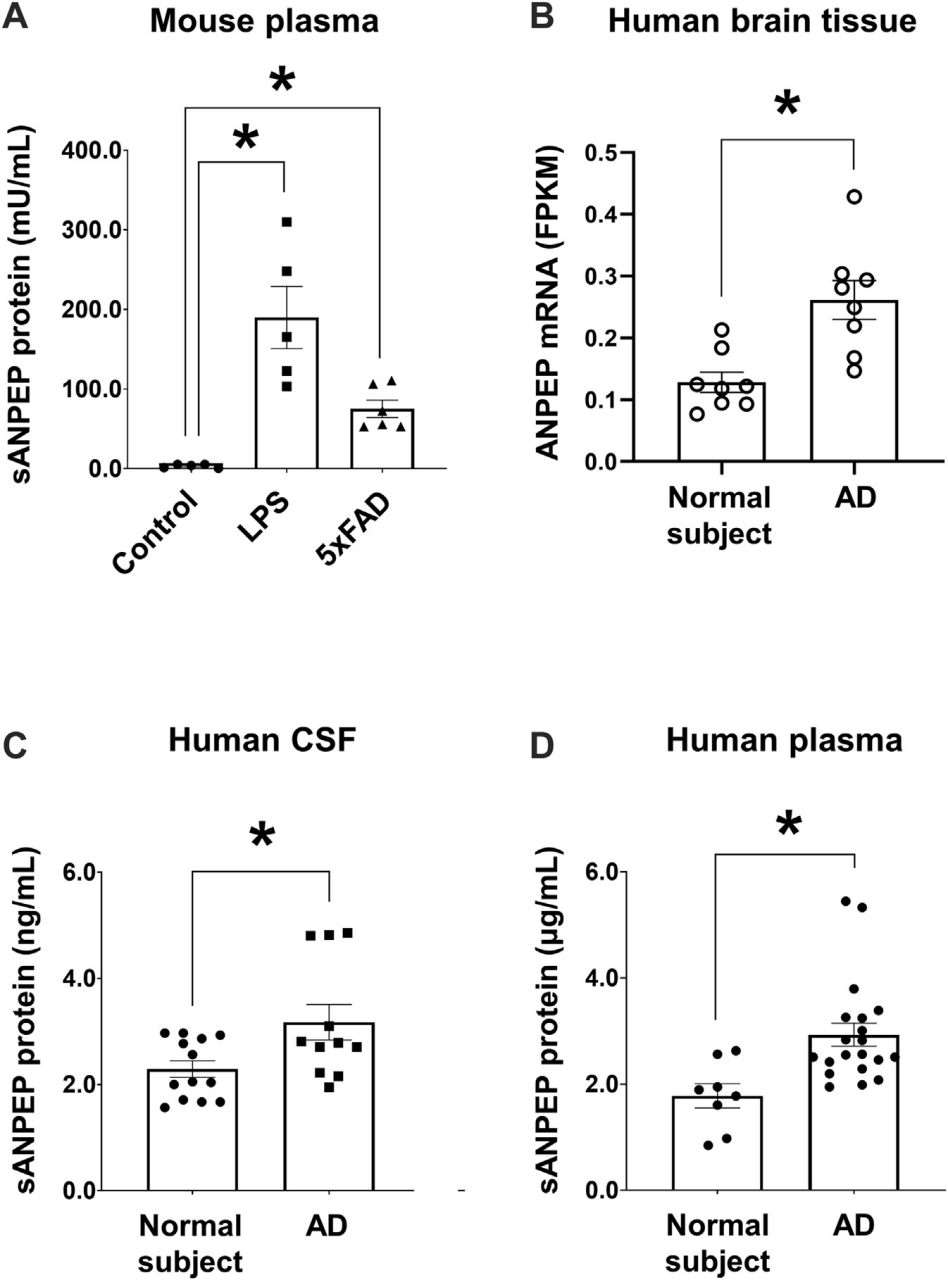
**sANPEP protein levels in body fluids and ANPEP mRNA expression in the brain tissue**. *A*, ELISA was performed to measure sANPEP protein levels in mouse plasma following LPS injection or the AD model (5xFAD). Control, n = 5; LPS, n = 5; 5xFAD, n = 6. *B*, human ANPEP mRNA levels in the brain of healthy controls and patients with AD were measured using transcriptome sequencing. Normal subject, n = 8; AD, n = 8. *C* and *D*, ELISA was used to determine the human sANPEP protein levels in the cerebrospinal fluid (CSF) (normal subject, n = 13; AD, n = 11) or plasma (normal subject, n = 8; AD, n = 20) of healthy controls and patients with AD. All data are presented as the mean ± SEM. One-way ANOVA was followed by (*A*) Tukey’s post hoc test and (*B,C*, and *D*) unpaired *t* test. *∗p* < 0.05. AD, Alzheimer’s disease; ANPEP, alanyl aminopeptidase; LPS, lipopolysaccharide; sANPEP, soluble form of ANPEP.

Proteins secreted from astrocytes are often found in the body fluids of animal models and in patients with neuroinflammation-related CNS disorders such as AD (46, 47, 48). The levels of sANPEP protein in the plasma of the mouse models of LPS (i.p.)-injected neuroinflammation and AD (5xFAD) were measured (Fig. 2*A*). sANPEP protein levels in the plasma of both neuroinflammation-related mouse models were significantly higher than those in the control mice. Previously, sANPEP was implicated as a biomarker for various diseases (25, 26, 27). Accordingly, we tested whether sANPEP could be utilized as a novel biomarker for neuroinflammation and neurodegenerative diseases in patients. We evaluated the levels of ANPEP mRNA in the brain tissues and sANPEP protein in the CSF and plasma samples (Fig. 2, *B* and *C*). ANPEP mRNA was significantly upregulated in AD brain tissues (Fig. 2*B*), consistent with an RNA sequencing analysis in a previous study (49), and sANPEP protein level was significantly higher in the CSF and plasma of AD patients than in healthy controls (Fig. 2*C*). We evaluated the potential confounding effect of age on the CSF and plasma levels of sANPEP (CSF*, t* = 2.221, *p* = 0.041; plasma, *t* = 0.9129, *p* = 0.377). As the two groups (patients and healthy controls) for the CSF study differed significantly in age, it was necessary to investigate the change in effect size when the age effect was removed from the measures. We assessed the effect of age by performing two stages of statistical analysis sequentially. First, the effect size was calculated using univariate ANOVA (IV, group; DV, human CSF) without the age variable (*η*^*2*^ = 22.4%, *p* = 0.02). The effect size was then calculated using analysis of covariance (IV, group; DV, human CSF), with age as a covariate (*η*^*2*^ = 21.5%, *p* = 0.026). We concluded that the CSF levels of sANPEP were still significantly different between the two groups, although the effect size decreased by 0.9%, when age was controlled. These data indicate that sANPEP may be a potential biomarker of AD.

### sANPEP Activated Microglia *via* the Ang IV–AT1R Ais

**Fig. 3:**
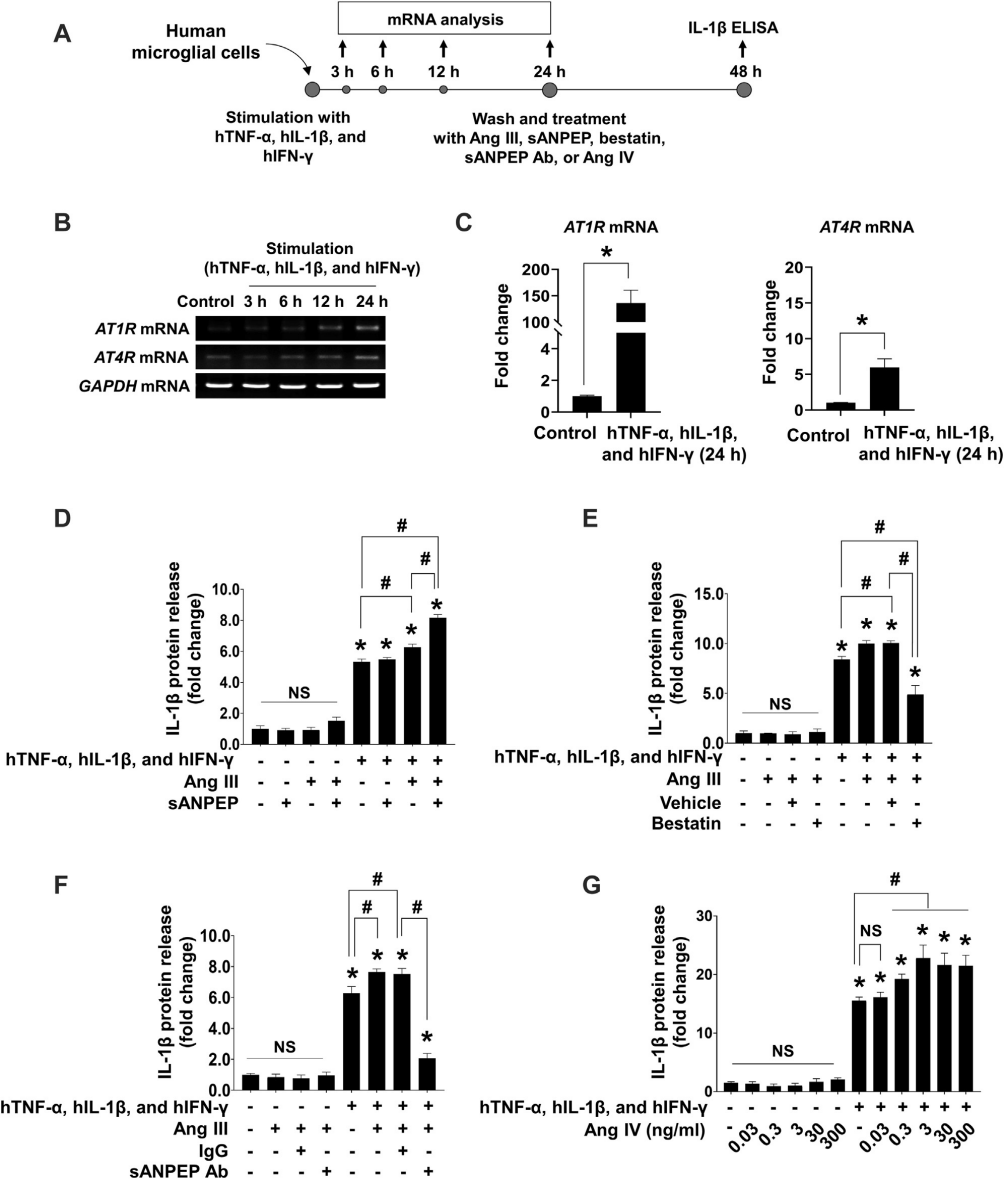
**Role of sANPEP in angiotensin-induced microglial activation**. *A*, scheme of the experiments. Human microglial cells were stimulated with human TNF-α, IL-1β, and IFN-γ (10 ng/ml each). For analysis of AT1R and AT4R mRNA expression, total RNA was collected at the indicated time points after stimulation. For measurement of IL-1β protein release, the cells were washed thoroughly five times using 1× HBSS at 24 h after stimulation and replenished with serum-free human microglia medium containing Ang III, sANPEP, vehicle, bestatin, IgG, neutralizing antibody for sANPEP (sANPEP Ab), or Ang IV. After 24 h, the conditioned medium was collected, and the release of IL-1β was analyzed using ELISA. *B*, time course of AT1R and AT4R mRNA expression following stimulation as determined by using conventional RT–PCR. *C*, fold changes of AT1R and AT4R mRNA expression at 24 h after stimulation as measured using real-time PCR. Data are mean ± SEM (n = 3 replicates per group). Unpaired *t* test. *∗p* < 0.05 *versus* unstimulated human microglial cells (control). *D*, effect of Ang III (30 nM) and sANPEP (100 ng/ml) on microglial IL-1β release (n = 5 replicates per group). Human microglial cells were stimulated with human TNF-α, IL-1β, and IFN-γ (10 ng/ml each). *E*, effect of sANPEP inhibitor (bestatin, 100 ng/ml) on Ang III (30 nM)-induced IL-1β release in the microglial cells (n = 5 replicates per group). Human microglial cells were stimulated with human TNF-α, IL-1β, and IFN-γ (10 ng/ml each). *F*, effect of neutralizing antibody for sANPEP (sANPEP Ab, 100 ng/ml) on Ang III (30 nM)-induced IL-1β release in the microglial cells (n = 5 replicates per group). Rat IgG was used as a control. Human microglial cells were stimulated with human TNF-α, IL-1β, and IFN-γ (10 ng/ml each). *G*, proinflammatory effect of Ang IV in the microglial cells (n = 5 replicates per group). Human microglial cells were stimulated with human TNF-α, IL-1β, and IFN-γ (10 ng/ml each). *D*–*G*, data are presented as the mean ± SEM using one-way ANOVA followed by Tukey’s post hoc test. ∗*p* < 0.05, *versus* the no-treatment control; ^*#*^*p* < 0.05. Ang III, angiotensin III; AT1R, angiotensin type 1 receptor; AT4R, angiotensin type 4 receptor; HBSS, Hank’s balanced salt solution; IFN-γ, interferon gamma; IgG, immunoglobulin G; IL-1β, interleukin 1β; NS, not significant; sANPEP, soluble form of aminopeptidase N; TNF-α, tumor necrosis factor alpha.

Next, we explored the regulatory role of sANPEP in Ang-induced neuroinflammation. As the actions of Ang are dependent on the expression of cognate receptors on target cells (50), we evaluated the expression of major Ang receptors in human microglial cells and astrocytes after proinflammatory stimulation (Fig. 3, *A*–*C*). Our data revealed low expression levels of AT1R and AT4R in unstimulated microglial cells. Although the expression of both AT1R and AT4R was upregulated at 6 to 24 h after stimulation (conventional RT–PCR; Fig. 3*B*), the upregulation of AT1R expression was dominant relative to that of AT4R (quantitative PCR; Fig. 3*C*). To elucidate the role of sANPEP in microglial activation, we treated human microglial cells with inflammatory stimuli and Ang III in the absence or the presence of recombinant sANPEP and measured IL-1β production (Fig. 3*D*). Along with inflammatory stimulation, the sANPEP treatment enhanced Ang III-induced IL-1β protein release, as measured using ELISA. Treatment of stimulated microglial cells with Ang III alone increased IL-1β production, which might have been because of endogenous angiotensinases, as their levels increased under inflammatory stimulation (supplemental Fig. S6). Ang III-induced IL-1β production in stimulated microglial cells was reduced by an sANPEP inhibitor (bestatin) (Fig. 3*E*) and neutralizing monoclonal antibody against ANPEP (Fig. 3*F*). In addition, Ang IV (from 0.3 to 300 ng/ml) treatment augmented IL-1β release from stimulated microglia (Fig. 3*G*). Since sANPEP may act as a ligand for G protein–coupled receptor (GPCR) (51) as well as a peptidase, we assessed the expression of B1R in human microglial cells and conducted an additional *in vitro* assay using a B1R antagonist. We observed very low levels of B1R mRNA expression in human microglial cells (supplemental Fig. S7*A*). The pharmacological antagonist of B1R did not influence IL-1β release significantly on induction by sANPEP (supplemental Fig. S7*B*). Therefore, the effect of GPCR engagement on sANPEP was excluded from our study. Collectively, these results indicate that sANPEP converts Ang III to Ang IV, which, in turn, acts on the upregulated AT1R expression in stimulated microglia to promote proinflammatory responses.

### sANPEP Promoted Neuroinflammation *in vivo*

**Fig. 4:**
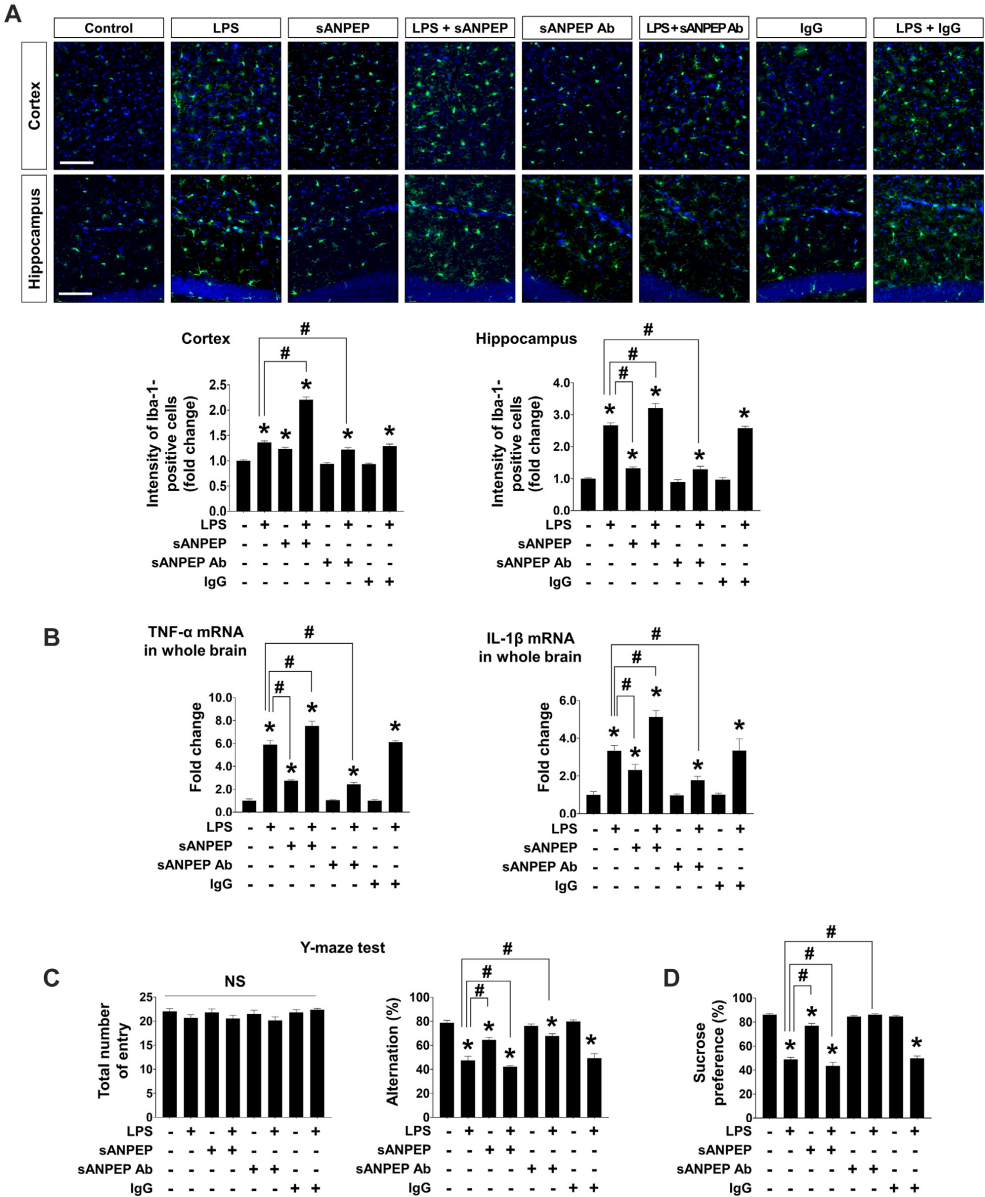
**sANPEP facilitates LPS-induced neuroinflammation**. *A*, immunofluorescence analysis of microglial activation in brain sections. Brain (cortex and hippocampus) sections were immunostained with anti-Iba-1 antibodies (*green*) 24 h after intraperitoneal LPS injection (1 mg/kg). Nuclei were stained with DAPI (*blue*). The scale bar represents 100 μm. Microglial activation was quantified as fold change of fluorescence intensity of Iba-1 compared with that in vehicle-injected group (n = 6 mice in each group). *B*, expression of proinflammatory cytokines (TNF-α and IL-1β) in the whole brain extracts of the mice (n = 6 mice in each group) was assessed using real-time PCR. *C*, Y-maze spontaneous alternation test (n = 6 mice in each group). *Left*, total number of entry. *Right*, spontaneous alternation test. *D*, sucrose preference test (n = 6 mice in each group). All data are presented as the mean ± SEM using one-way ANOVA followed by Tukey’s post hoc test. ∗*p* < 0.05 *versus* the no-injection control; ^#^*p* < 0.05, *versus* LPS. DAPI, 4′,6-diamidino-2-phenylindole; IL-1β, interleukin 1β; LPS, lipopolysaccharide; NS: not significant; sANPEP, soluble form of ANPEP; TNF-α, tumor necrosis factor alpha.

To determine the role of sANPEP in neuroinflammation *in vivo*, mice were i.p. injected with LPS (1 mg/kg) and an i.c.v. infusion of recombinant ANPEP (3 μg) or neutralizing monoclonal antibody against ANPEP (sANPEP antibody, 6 μg). The effects of sANPEP or ANPEP antibody on LPS-induced microglial activation, expression of proinflammatory cytokines, and behavioral deficits were assessed (Fig. 4). Immunohistochemistry data revealed that the intensity of Iba-1, which represented microglial activation, was significantly increased in the cortex and hippocampal region of i.p. LPS-injected mouse brain and further enhanced by i.c.v. infusion of sANPEP. The LPS-induced microglial activation was attenuated by i.c.v. infusion of neutralizing antibodies for sANPEP (Fig. 4*A*). The effect sizes of systemic administration of LPS and the antibody were partly mild and brain region specific. This may be due to regional differences in susceptibility to LPS (52). Further experiments are required to fully elucidate their physiological relevance. Systemic LPS-induced mRNA expression of proinflammatory cytokines, loss of acute spatial memory, and anhedonia (lack of sucrose preference) were significantly enhanced by sANPEP infusion (Fig. 4, *B*–*D*). Neuroinflammation and behavioral deficits in systemic LPS-injected mice were clearly alleviated after i.c.v. infusion of neutralizing antibodies (Fig. 4, *B*–*D*). These results support the proinflammatory role for sANPEP *in vivo*.

## Discussion

In this study, we profiled the secretome of stimulated human astrocytes. Only a few previous studies have reported the secretome profiles of human astrocytes owing to the limited use of human brain tissues (53, 54, 55, 56). Cells isolated from mice are the most frequently used *in vitro* models for disease research owing to their ease of cultivation and manipulation. However, crucial differences in cells derived from humans and mice have been reported (57, 58), such as the morphological features, mitochondrial physiology, and oxidative stress susceptibility. In terms of astrocytes, human astrocytes express TLR4 and MD2 but not CD14, whereas mouse astrocytes express all three; thus, striking differences occur between human and mouse astrocytes in the use of Toll-like receptor/IL-1R and subsequent downstream signaling and immune activation (59). In this study, stimulation with a combination of TNF-α, IL-1β, and IFN-γ successfully induced the expression of inducible NO synthase and proinflammatory cytokines in human astrocytes (supplemental Fig. S1). GO enrichment and KEGG pathway analyses of the secretome of stimulated astrocytes established a reactive phenotype of astrocytes associated with neuroinflammatory activation (Figs. 1 and S4). Therefore, our secretome data have important implications for translational research on human CNS diseases related to neuroinflammation.

In the current study, we collected secreted proteins from astrocytes cultured in media that did not contain any supplements to avoid the detection of contaminating serum proteins, and so on, such as growth factors and albumin. However, because of cellular stress and cell death, culturing the cells under serum starvation conditions can affect secretome composition (60). To overcome this limitation, alternative secretomics approaches compatible with cell culture in the presence of serum or supplements have been developed, such as stable isotope labeling by amino acids in cell culture (61), secretome protein enrichment with click sugars (SPECS) method (62), high-performance secretome protein enrichment with click sugars (hiSPECS) method (39), combination of bioorthogonal noncanonical amino acid tagging, and pulsed-stable isotope labeling by amino acids in cell culture (63), and various proximity labeling systems (64, 65).

Here, we propose that sANPEP can serve as a putative biomarker for neuroinflammation. We observed higher levels of sANPEP in the plasma of LPS-injected and 5xFAD mice (Fig. 2*A*). In the brain tissue from AD patients, mRNA expression of ANPEP was upregulated (Fig. 2*B*). A significant increase in sANPEP was detected in the CSF and plasma of patients with AD (Fig. 2*C*). ANPEP was originally identified as a cell surface protein for myeloid cell markers (66) and is now reported to be expressed in a variety of cells, such as epithelial cells, neural cells, fibroblasts, and activated endothelial cells (67). In the brain, ANPEP has been used as a pericyte marker (68, 69), although its expression has also been observed in glial cells, such as astrocytes and microglia (70, 71, 72). Membrane ANPEP can be shed in a soluble form under certain inflammatory conditions. The expression of metalloproteases MMP-9 (26) and MMP-14 (23) is upregulated at the site of inflammation, and they represent potential enzymes that cleave membrane ANPEP. In our study, the cellular origin of the increased sANPEP levels *in vivo* likely was not only limited to astrocytes but also included other cell types, such as mural cells and infiltrating myeloid cells. Nevertheless, the increase in sANPEP levels may be closely associated with glial activation and neuroinflammation. Astrocytes are the most abundant glial cell type in the brain, and they are in a prolonged activation state in pathological brains, which is referred to “reactive gliosis” (73, 74). Metalloproteases are upregulated in reactive astrocytes and pathological brains (75). Therefore, the heightened levels of sANPEP may represent a state of neuroinflammation. A previous study suggested that patients with AD had lower vascular levels of membrane ANPEP/CD13 in brain mural cells, and this reduction was correlated with lower cognitive performance (76). They investigated the amounts of the membrane form of CD13 protein in human and murine brain microvessels by Western blot analysis. Moreover, immunofluorescence analyses indicated that ANPEP/CD13 protein was localized in the membrane of brain mural cells. In our study, we observed an increase in the soluble form of the ANPEP/CD13 protein in the plasma and CSF of AD mice and patients. Under pathological conditions, membrane ANPEP is cleaved by MMP proteases (23). Therefore, a reduction in membrane ANPEP levels and an increase in soluble ANPEP levels may be associated with AD pathophysiology.

Our study showed that the role of sANPEP in neuroinflammation was dependent on its enzymatic activity, in which Ang III is converted to Ang IV. Moreover, we demonstrated that (1) sANPEP treatment alone did not affect microglial IL-1β release; (2) cotreatment with sANPEP and Ang III enhanced IL-1β release in stimulated microglia (Fig. 3*D*); and (3) Ang IV treatment increased IL-1β release in stimulated microglia (Fig. 3*G*). Reports have also shown that sANPEP plays roles that are independent of its enzyme activity; for example, sANPEP acts as a ligand for a GPCR, which triggers the activation of the JNK, Src, and NF-κB pathways to upregulate the expression of cytokines and chemokines, such as IL-1β, IL-6, and MCP-1, thereby augmenting the infiltration of monocytes or macrophages seen in rheumatoid arthritis (28). Therefore, the mode of action of sANPEP may be context dependent.

**Fig. 5:**
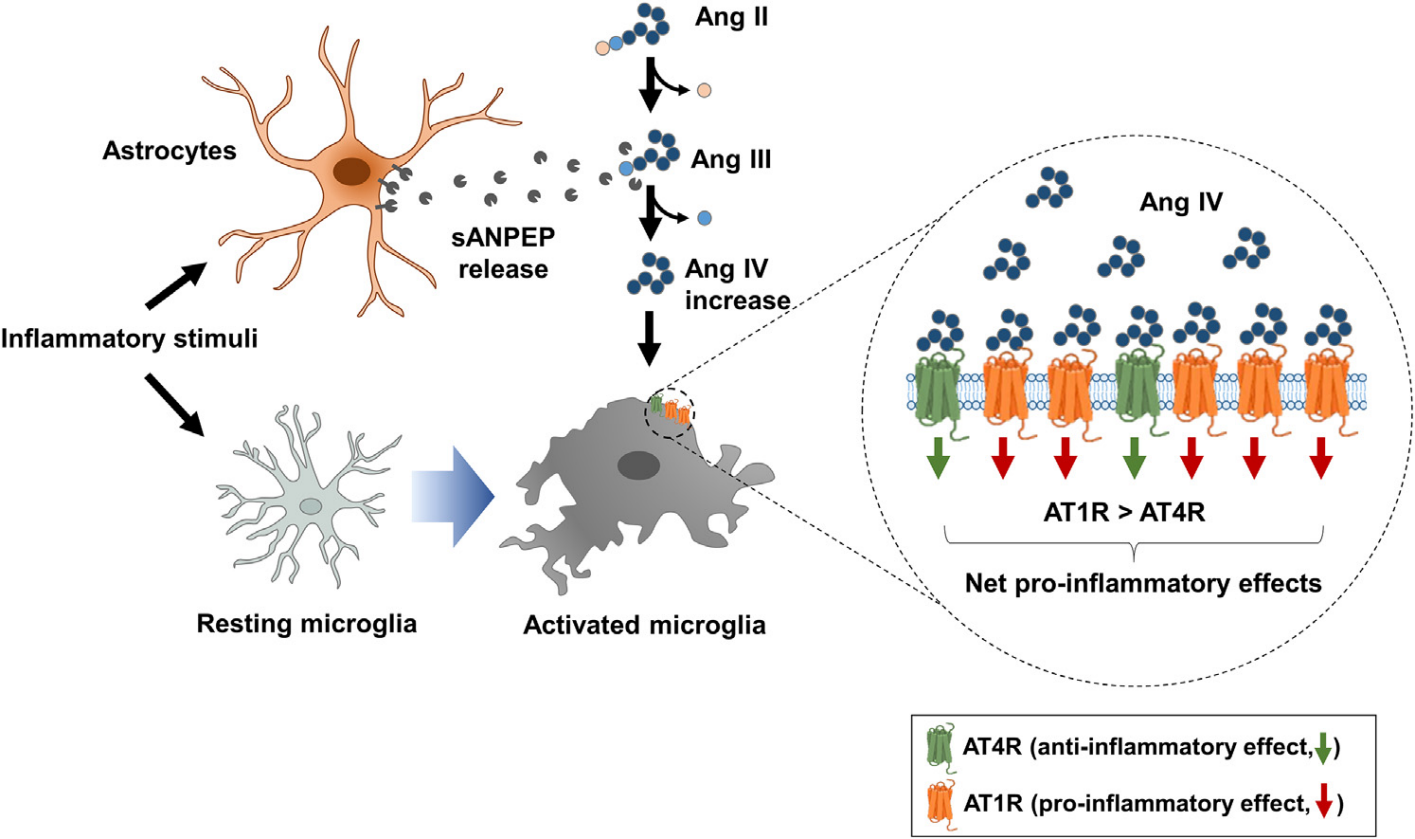
**Summary of the role of sANPEP in facilitating microglial activation.** Within a neuroinflammatory milieu, sANPEP is released from activated astrocytes. Then, sANPEP cleaves Ang III to generate Ang IV. Moreover, the expression of AT1R is dominantly upregulated in stimulated microglia under the same conditions. Ang IV generated by sANPEP interacts with proinflammatory AT1R to promote microglial activation. Ang III, angiotensin III; AT1R, angiotensin type 1 receptor; sANPEP, soluble form of aminopeptidase N.

This study suggests that astrocytic sANPEP, a component of the brain RAS, is a proinflammatory regulator of microglia (Fig. 5). Ang peptides in the brain regulate various physiological (29) and pathological processes, such as microglial activation (77, 78) and vascular inflammation (79, 80). The functional outcomes of Ang peptides depend on the activity and distribution of various enzymes and receptors (81). These actions have also been implicated in several CNS disorders, such as AD, Parkinson’s disease, multiple sclerosis, and bipolar disorder (82). The alteration in the expression of genes involved in RAS and activity under neuroinflammatory conditions may be associated with the pathogenesis of these CNS disorders. Therefore, the upregulation of sANPEP in human astrocytes and biofluids associated with neuroinflammatory conditions suggests the diagnostic potential of sANPEP, whereas its enzymatic activity in generating Ang IV to promote microglial activation implies the therapeutic potential of sANPEP as a drug target for neuroinflammatory diseases.

## Data Availability

The data generated or analyzed during the current study are available from the corresponding author upon reasonable request. The MS proteomics data have been deposited at the ProteomeXchange Consortium (http://www.proteomexchange.org) *via* the MASSIVE repository (MSV000090205) with the dataset identifier PXD036257.

## Supplemental data

This article contains supplemental data.

## Conflict of interest

The authors declare no competing interests.
