# Supplementary material for: Soluble ANPEP Released From Human Astrocytes as a Positive Regulator of Microglial Activation and Neuroinflammation: Brain Renin–Angiotensin System in Astrocyte–Microglia Crosstalk
Source: Mol Cell Proteomics. 2022 Oct 8;21(11):100424. doi: 10.1016/j.mcpro.2022.100424 (PMC9650055; doi:10.1016/j.mcpro.2022.100424)
Supplement: Supplemental figures and tables [file mmc1.docx]

*Supplemental Data for*

**Soluble ANPEP released from human astrocytes as a positive regulator of microglial activation and neuroinflammation: Brain renin–angiotensin system in astrocyte–microglia crosstalk**

Jong-Heon Kim^1^, Afridi Ruqayya^2^, Eunji Cho^3^, Jong Hyuk Yoon^3^, Yong-Hyun Lim^4^, Ho-Won Lee^1,5^, Hoon Ryu^6,7,8^, and Kyoungho Suk^1,2*^

^1^Brain Science & Engineering Institute, Kyungpook National University, Daegu, Republic of Korea; ^2^Department of Biomedical Science, School of Medicine, Kyungpook National University, Daegu, Republic of Korea; ^3^Neurodegenerative Diseases Research Group, Korea Brain Research Institute, Daegu, Republic of Korea; ^4^Center of Self-Organizing Software-Platform, Kyungpook National University, Daegu, Republic of Korea; ^5^Department of Neurology, Kyungpook National University Chilgok Hospital, School of Medicine, Kyungpook National University, Daegu, Republic of Korea; ^6^Center for Neuromedicine and Neuroscience, Brain Science Institute, Korea Institute of Science and Technology, Seoul, Republic of Korea; ^7^VA Boston Healthcare System, Boston, MA, USA; ^8^Boston University Alzheimer's Disease Center and Department of Neurology, Boston University School of Medicine, Boston, MA, USA

*Corresponding Author: Kyoungho Suk, Ph.D.

Department of Pharmacology, School of Medicine, Kyungpook National University, 680 Gukchaebosang Street, Joong-gu, Daegu 41944, Republic of Korea

E-mail: [ksuk@knu.ac.kr](mailto:ksuk@knu.ac.kr); Telephone: +82-53-420-4835; Fax: +82-53-256-1566

Table of Contents

Figure S1 ……………………………………………….. Page 4

Figure S2 ……………………………………………….. Page 5

Figure S3 ……………………………………………….. Page 6

Figure S4 ……………………………………………….. Page 7

Figure S5 ……………………………………………….. Page 8

Figure S6 ……………………………………………….. Page 9

Figure S7 ……………………………………………….. Page 10

Supplemental Tables …………………………………..... Page 11


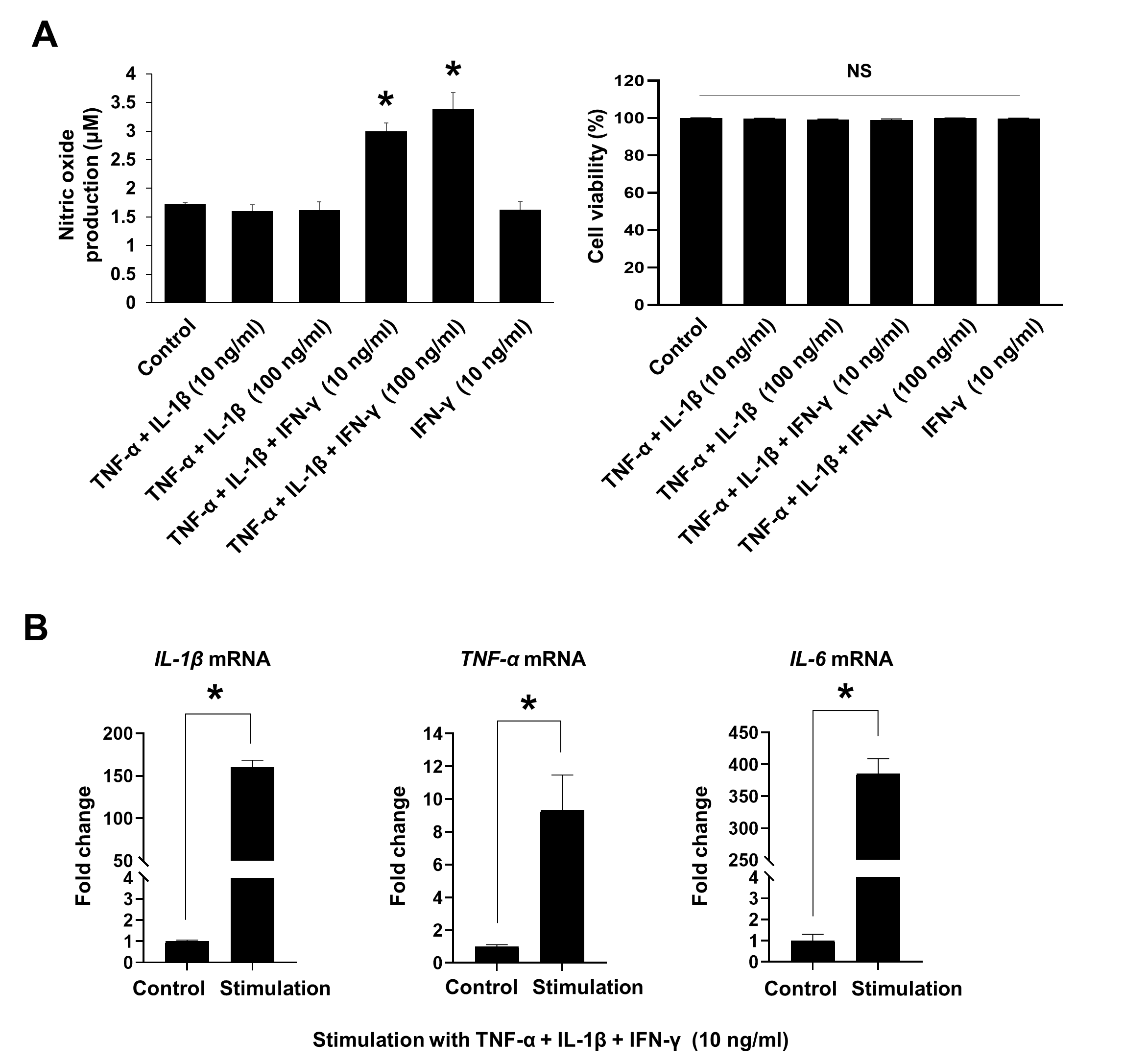


**Figure S1. Activation of human astrocytes by proinflammatory cytokines.**

A. Nitric oxide production in human astrocytes after stimulation with proinflammatory cytokines (n = 3 replicate wells per group). Nitric oxide concentration was assessed after 48 h of stimulation (*left*). Cell viabilities were measured using the MTT assay (*right*). NS: not significant.

B. Proinflammatory cytokine mRNA levels in human astrocytes stimulated with human TNF-α, IL-1β, and IFN-γ (10 ng/ml each) for 24 h (n = 3 replicate wells per group).

All data are presented as the mean ± SEM using one-way analysis of variance followed by Tukey’s *post hoc* test. *^*^P* < 0.05 versus unstimulated human astrocytes (control).


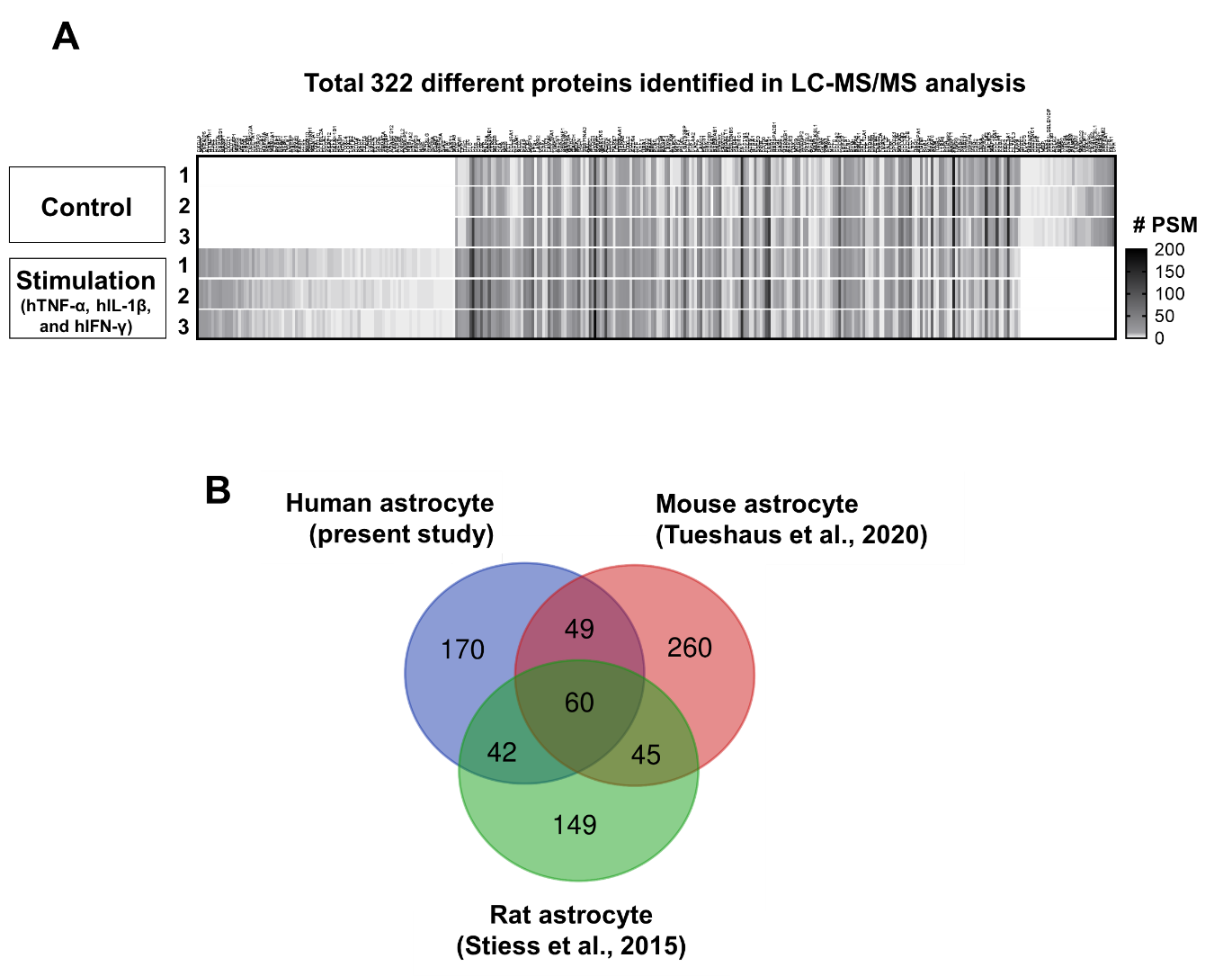


**Figure S2. Intensity-based heatmap of the 322 proteins found in the human astrocyte secretome (A) and comparison of our data with those of rodent astrocytes (B).** (A) Heatmap indicating the number of peptide spectral match (PSM) of the 322 secreted proteins identified using LC-MS/MS analysis of three replicates of astrocyte-conditioned media under control or stimulation conditions. (B) Comparison between our data obtained from human astrocytes and those obtained from rodent astrocytes reported in the literature.


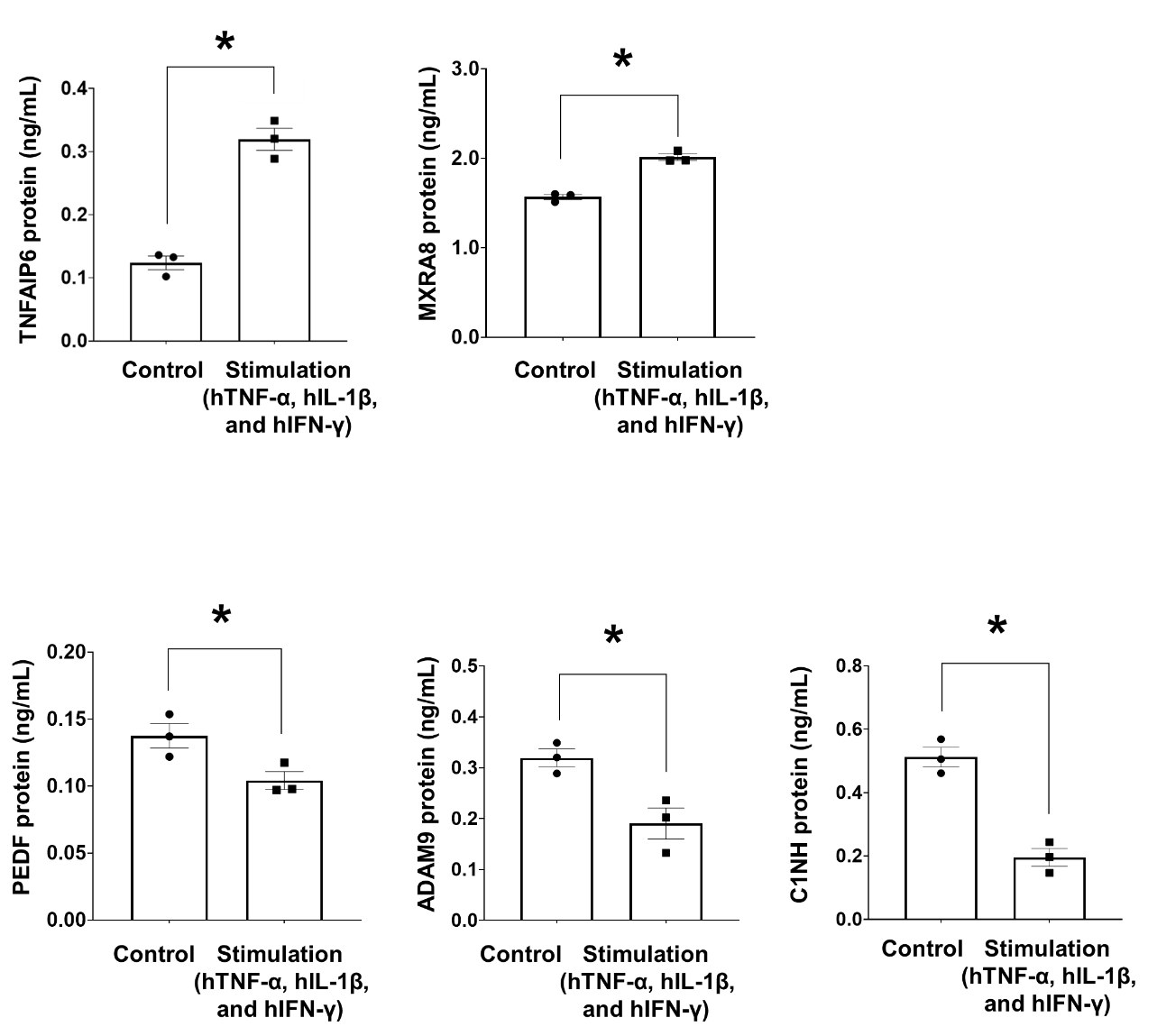


**Figure S3. ELISA-based validation of differential protein expression identified using the LC/MS-MS analysis.** Levels of significantly altered secreted proteins from the LC/MS-MS analysis were measured using ELISA kits for human TNFAIP6 (Mybiosource), MXRA8 (Cusabio), PEDF (Cloud-clone), ADAM9 (R&D systems), and C1NH (R&D systems) according to the manufacturer’s instructions. Data are presented as the mean ± SEM (n = 3 replicate wells per group), using paired *t* test. *^*^P* < 0.05.


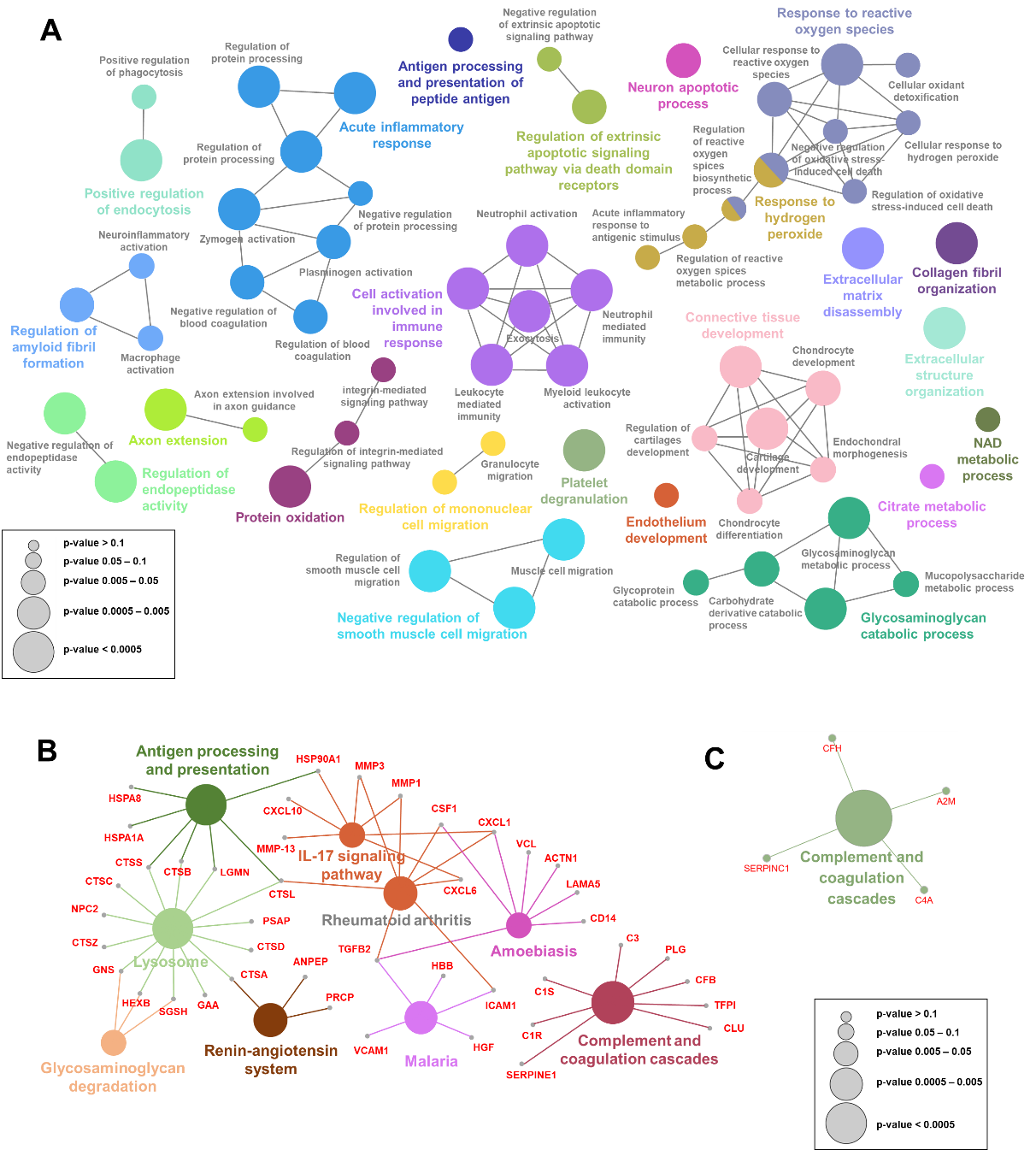


**Figure S4. Representative functional group network views for Gene Ontology (GO) terms and KEGG pathways.**

A. GO term enrichment (biological processes) analysis of differentially expressed proteins.

B. KEGG pathways analysis of upregulated proteins in the conditioned media of stimulated human astrocytes.

C. KEGG pathways of downregulated proteins in the conditioned media of stimulated human astrocytes.

Terms are functionally grouped based on shared genes (kappa score) and shown in different colors.


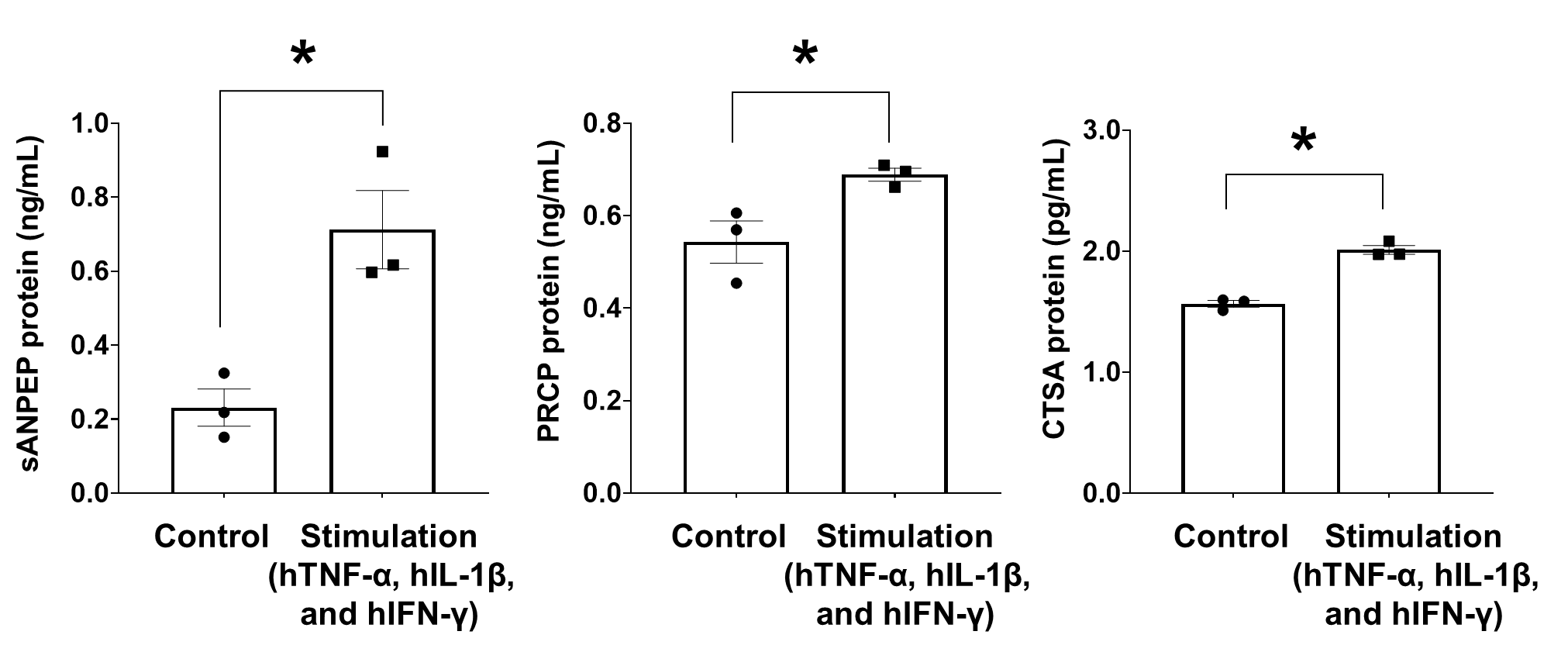


**Figure S5. Increase in the levels of proteins associated with brain RAS in human astrocytes following stimulation with proinflammatory factors.** Human astrocytes were stimulated with human TNF-α, IL-1β, and IFN-γ (10 ng/ml each) for 24 h. Then, the cells were washed thoroughly five times using 1× HBSS, and serum-free human astrocyte medium was replenished. After 24 h, conditioned medium was collected. Levels of proteins in the conditioned medium were measured by ELISA kits for human ANPEP (R&D systems), PRCP (Cloud-clone), and CTSA (Mybiosource). Data are presented as the mean ± SEM (n = 3 replicate wells per group), using paired *t* test. *^*^P* < 0.05.


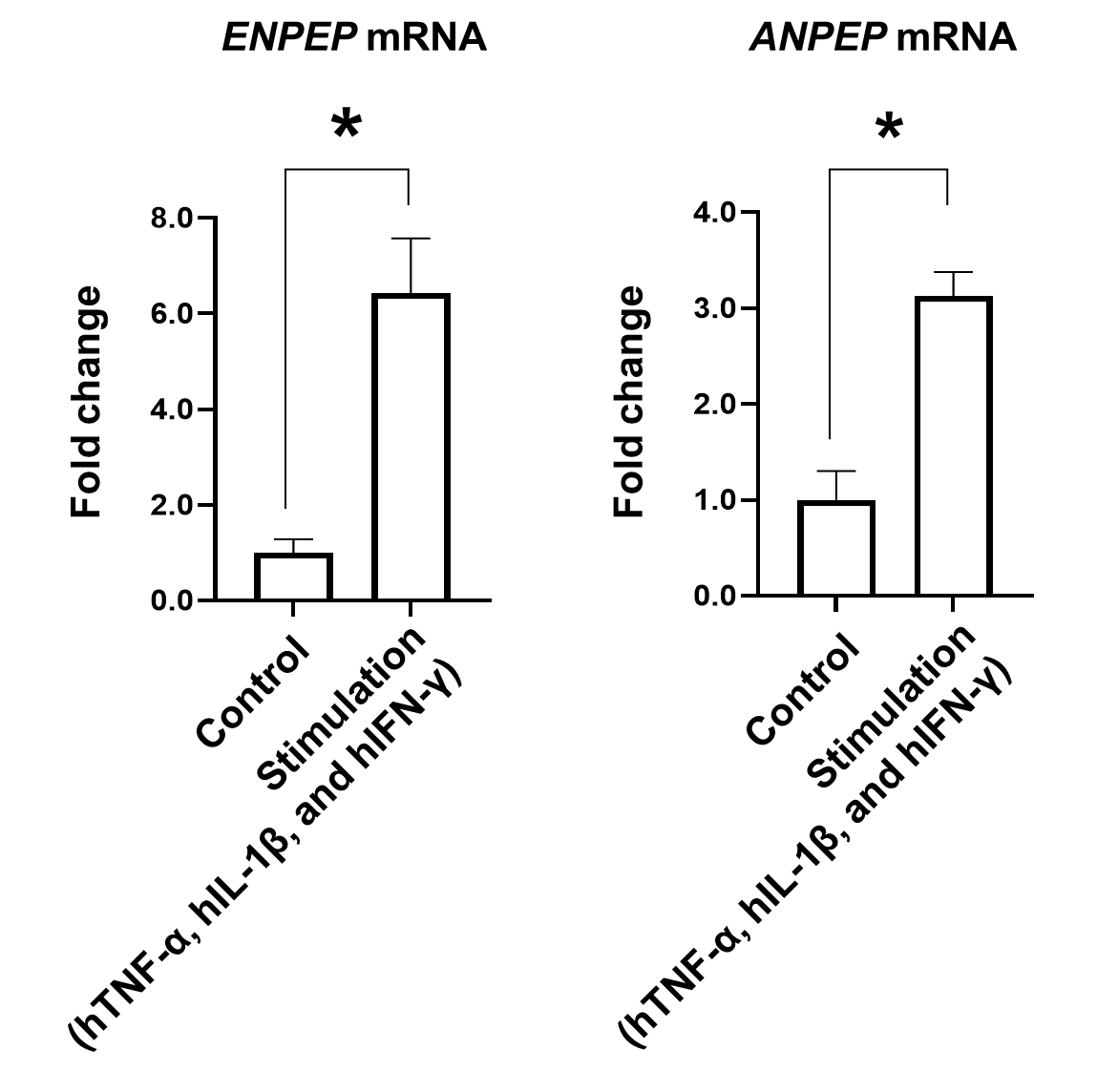


**Figure S6. Increase in ENPEP and ANPEP mRNA expression in human microglial cells.** Human microglial cells were stimulated with human TNF-α, IL-1β, and IFN-γ (10 ng/ml each) for 24 h. The total extracted RNA was subjected to real-time PCR. Data are presented as the mean ± SEM (n = 3 replicate wells per group), using paired *t* test. *^*^P* < 0.05.

**
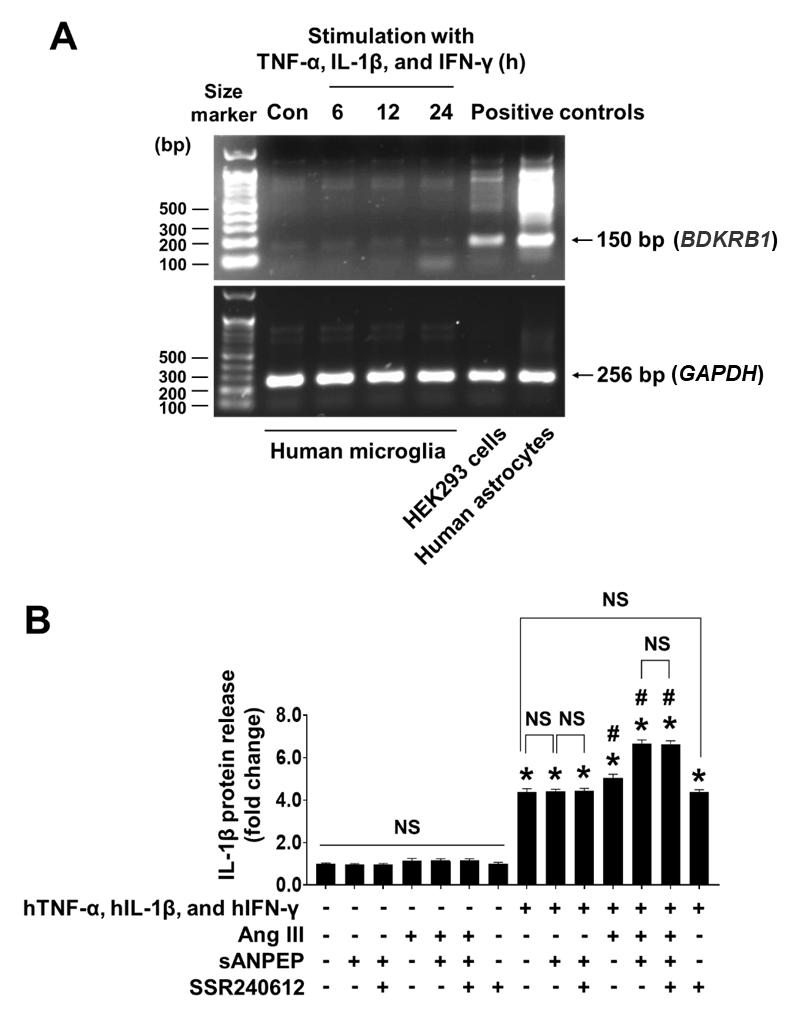
**

**Figure S7. Expression of bradykinin receptor B1R (BDKRB1) mRNA (A) and effects of SSR240612 (a B1R antagonist) on sANPEP-induced IL-1β release in human microglial cells (B).** (A) Human microglial cells were stimulated with human TNF-α, IL-1β, and IFN-γ (10 ng/ml each) for 6, 12, or 24 h. The total extracted RNA was subjected to conventional RT-PCR. HEK293 human embryonic kidney cells and human astrocytes were used as positive controls for B1R expression. Con: unstimulated human microglia. (B) Effects of SSR240612 (50 μM) (a B1R antagonist) on microglial IL-1β release induced by Ang III (30 nM) and sANPEP (100 ng/ml) (n = 5 replicates per group). Microglial cells were treated with SSR240612 for 1 h before cytokine stimulation. All data are presented as the mean ± SEM using one-way analysis of variance (ANOVA) followed by Tukey’s post hoc test. *P < 0.05, versus the no-treatment control; #P < 0.05, versus cytokine stimulation. NS: not significant.

Supplemental Tables

**Table S1. DNA sequences of the primers used for RT-PCR and real-time PCR**

| Gene | RT-PCR | Primer sequences | GenBank accession no. |
| --- | --- | --- | --- |
| Mouse *tnf-α* | Real-time | F: 5′-TCACACTCAGATCATCTTCTC-3′  R: 5′-ATGAGATAGCAAATCGGCTG-3′ | NM_013693 |
| Mouse *il-1β* | Real-time | F: 5′-CTTTGAAGAAGAGCCCATCC-3′  R: 5′-TTTGTCGTTGCTTGGTTCTC-3′ | NM_008361 |
| Human *AT1R* | Conventional,  Real-time | F: 5’-GGAAACAGCTTGGTGGTGAT-3’  R: 5’-GCAGCCAAATGATGATGCAG-3’ | NM_000685.5 |
| Human *AT4R* | Conventional,  Real-time | F: 5’-GGACAGATCGCCATTGTTGC-3’  R: 5’-AGCGGTGTATTGCTCATCCC-3’ | NM_005575.3 |
| Human *IL-1β* | Real-time | F: 5′-ACGCTCCGGGACTCACAGCA-3′  R: 5′-TGAGGCCCAAGGCCACAGGT-3′ | NM_000576 |
| Human *TNF-α* | Real-time | F: 5′-GCCCAGGCAGTCAGATCATCT-3′  R: 5′-TTGAGGGTTTGCTACAACATGG-3′ | NM_000594 |
| Human *IL-6* | Real-time | F: 5′-GCAACACCAGGAGCAGCC-3′  R: 5′-AACTCCTTCTCCACAAGCGC-3′ | NM_000600 |
| Human *ENPEP* | Real-time | F: 5′-GTCCGGAGGTGTTTCGAGTA-3′  R: 5′-CGTTCTCCGTGTAGGTGGTT-3′ | NM_001977 |
| Human *ANPEP* | Real-time | F: 5′-GTGCTCCAGTGGTTCACAGA-3′  R: 5′-GGCTCACAGGCAGAGAGAAC-3′ | NM_001150 |
| Human *BDKRB1* | Conventional | F: 5′-TGCCAACATTTATCATCTCC-3′  R: 5′-AAGCCCAAGACAAACACC-3′ | NM_001386007.1 |
| Mouse *gapdh* | Real-time | F: 5’-CTCATGACCACAGTCCATGC-3’  F: 5’-TTCAGCTCTGGGATGACCTT-3’ | NM_008084 |
| Human *GAPDH* | Conventional | F: 5′-ACCACAGTCCATGCCATCAC-3′  R: 5′-TCCACCACCCTGTTGCTGTA-3′ | NM_001289745.2 |
|  | Real-time | F: 5′-GAAATCCCATCACCATCTTCC-3′  R: 5′-GAGGCTGTTGTCATACTTCTC-3′ |  |

**Table S2. Information on the brain tissue samples from healthy controls and patients with AD**

| **Case** | **Number** | **Age** | **Sex** | **Braak stage** |
| --- | --- | --- | --- | --- |
|  |  |  |  |  |
| Healthy | 1 | 87 | F |  |
|  | 2 | 86 | M |  |
|  | 3 | 87 | F |  |
|  | 4 | 89 | M |  |
|  | 5 | 78 | F |  |
|  | 6 | 82 | M |  |
|  | 7 | 73 | F |  |
|  | 8 | 89 | M |  |
| AD | 1 | 88 | M | VI |
|  | 2 | 82 | M | V |
|  | 3 | 79 | F | VI |
|  | 4 | 80 | F | V |
|  | 5 | 83 | M | VI |
|  | 6 | 77 | M | VI |
|  | 7 | 89 | F | V |
|  | 8 | 89 | M | IV |
|  |  |  |  |  |

F, female; M, male; AD, Alzheimer’s disease

*Braak stage

Stage I and II: either mild or severe alteration of the transentorhinal layer pre-alpha; Stage III and IV: a conspicuous effect on the layer pre-alpha in both the transentorhinal region and the actual entorhinal cortex, as well as mild involvement of the first Ammon's horn sector; Stage V and VI: the destruction of virtually all isocortical association areas.

**Table S3. Demographics and clinical characteristics of healthy controls and patients for CSF and plasma studies**

| CSF study | | |
| --- | --- | --- |
| Characteristic | Healthy (n = 13) | AD (n = 11) |
| Gender (M/F) | 7/6 | 3/8 |
| Age (years) | 67.14 ± 6.93 | 71.63 ± 8.51 ^**^ |
| MMSE score | 27.23 ± 2.46 | 16 ± 3.64 ^***^ |
| CDR score | 0.25 ± 0.15 | 1.31 ± 0.53 ^***^ |
| Education (years) | 11.45 ± 3.89 | 3.45 ± 2.99 ^**^ |
| BMI | 20.2 ± 1.12 | 24.85 ± 3.7 |

| Plasma study | | |
| --- | --- | --- |
| Characteristic | Healthy (n = 8) | AD (n = 20) |
| Gender (M/F) | 3/5 | 4/16 |
| Age (years) | 67.88 ± 6.17 | 70.33 ± 7.14 ^**^ |
| MMSE score | 29.12 ± 1.69 | 18.94 ± 6.76 ^***^ |
| CDR score | 0.16 ± 0.16 | 1 ± 0.577 ^***^ |
| Education (years) | 12.12 ± 3.58 | 5.5 ± 4.96 ^**^ |
| BMI | 24.12 ± 3.47 | 24.87 ± 3.4 |

M, male; F, female; MMSE, mini-mental state examination; CDR, clinical dementia rate; BMI, body mass index

Values are mean ± SD. ^**^*P* < 0.001 and ^***^*P* < 0.0001 vs. normal subjects.

**Table S4.** List of proteins identified in the LC-MS/MS analysis of the human astrocyte secretome. **See Excel File Table S4_List of identified proteins**

**Table S5.** Biological processes of upregulated secretory proteins in stimulated human astrocytes. **See Excel File Table S5_Biological processes**

**Table S6.** KEGG pathways of upregulated secretory proteins in stimulated human astrocytes. **See Excel File Table S6_KEGG pathways**

**Table S7.** Information of proteins identified. **See Excel File Table S7_Information of proteins identified.**
